# Lack of predictive capacity of pre-transplant anti-BK virus antibodies for post-transplant reactivation

**DOI:** 10.1007/s40620-022-01487-7

**Published:** 2022-12-02

**Authors:** Arturo Blazquez-Navarro, Toralf Roch, Patrizia Wehler, Ulrik Stervbo, Chris Bauer, Kerstin Wolk, Robert Sabat, Chantip Dang-Heine, Oliver Thomusch, Petra Reinke, Christian Hugo, Panagiota Zgoura, Richard Viebahn, Timm Westhoff, Michal Or-Guil, Nina Babel

**Affiliations:** 1 Center for Translational Medicine and Immune Diagnostics Laboratory, Medical Department I, Marien Hospital Herne, Ruhr-University Bochum, University Hospital of the Ruhr-University Bochum, Herne, North Rhine-Westphalia Germany; 2 BIH Center for Regenerative TherapiesBerlin Institute of Health, Charité–Universitätsmedizin Berlin, Corporate Member of Freie Universität Berlin, Humboldt-Universität Zu Berlin, Berlin, Germany; 3 Department of Infectious Diseases, West-German Centre for Infectious Diseases, University Hospital Essen, University Duisburg-Essen, Essen, North Rhine-Westphalia Germany; 4 Institute of Medical Immunology, Charité–Universitätsmedizin Berlin, Corporate Member of Freie Universität Berlin, Humboldt-Universität Zu Berlin, and Berlin Institute of Health, Berlin, Germany; 5 Albert-Ludwigs-Universität Freiburg, Klinik für Allgemein- und Viszeralchirurgie, Hugstetter Str. 55, Baden-Württemberg 79106 Freiburg, Germany; 6 Berlin Center for Advanced Therapies (BeCAT), Charité–Universitätsmedizin Berlin, Augustenburger Platz 1, 13353 Berlin, Germany; 7 Medizinische Klinik III - Bereich Nephrologie, Universitätsklinikum Carl Gustav Carus, Fetscherstraße 74, 01307 Dresden, Germany; 8 Chirurgische Klinik, Universitätsklinikum Knappschaftskrankenhaus Bochum, In Der Schornau 23-25, 44892 Bochum, Germany; 9 Institute of Medical Immunology, Charité – Universitätsmedizin Berlin, Charitéplatz 1, 10117 Berlin, Germany

BK virus (BKV) is a very common pathogen infecting up to 90% of the general population [1]. While usually innocuous, BKV reactivation is a frequent complication after renal transplantation leading to graft loss in 1–10% of cases [1]. Risk factors for reactivation include age, male sex, graft rejection and the use of calcineurin inhibitors [2]. However, it is still unclear whether pre-transplant BKV-reactive antibody levels of the transplant recipient are associated with BKV reactivation post transplant. This is in strong contrast with the case of cytomegalovirus, for which pre-transplant serostatus is an established marker. [3]

We characterized the kidney transplant recipients of a large, clinically well-characterised multi-centre cohort (*N* = 397) for the presence of serum IgG antibodies against the structural BKV protein VP1, using an ELISA assay (see Supplementary Methods and Fig. S1) [3, 4]. In parallel, BKV load in serum was monitored pre-transplant and then two weeks and one, two, three, six, nine and twelve months post-transplant, as published previously [3]. A total of 2092 samples were analysed for BKV load. Significant differences in quantitative variables were calculated employing the Mann–Whitney *U* test; correlations were assessed using Spearman's rank correlation coefficient.

Three hundred ninety-five (99.5%) patients had detectable pre-transplant anti-BKV antibodies, with a median [IQR] IgG concentration of 23 [13–38] µg/ml (Fig. 1A). BKV load over the detection limit (> 250 copies/mL) was observed in 196 (49.4%) patients, with a peak viral load of 1463 [731–7444] copies·mL^−1^ (Fig. 1B). Reactivations occurred at a median time of 63 [31–181] days post-transplant. Importantly, no association between anti-BKV IgG concentrations and reactivation was observed (no reactivation: 22 [14–38] µg/ml, reactivation: 25 [13–38] µg/ml; *P* = 0.886; Fig. 1C). We also evaluated whether the anti-BKV IgGs correlate with the height of viral load; no correlation was found (*ρ* = 0.00, *P* = 0.955; Fig. 1D). Interestingly, 33 patients (8.3%) had detectable BKV load before transplantation, with a median viral load of 770 [422–1442] copies·mL^−1^. These patients also had increased anti-BKV antibody concentrations (35 [17–53] vs. 22 [13–37] µg/ml; *P* = 0.010; Fig. 1E). Independently from this association, we observed that patients with early post-transplant BKV reactivation (< 60 days) had significantly higher anti-BKV antibody concentrations than patients with late reactivation (29 [16–45] vs. 22 [11–33] µg/ml; *P* = 0.007; Fig. 1F). Finally, there was no correlation between anti-BKV IgG and graft function one year post-transplant (*ρ* = 0.00, *P* = 0.950; Fig. 1G).

**Fig. 1:**
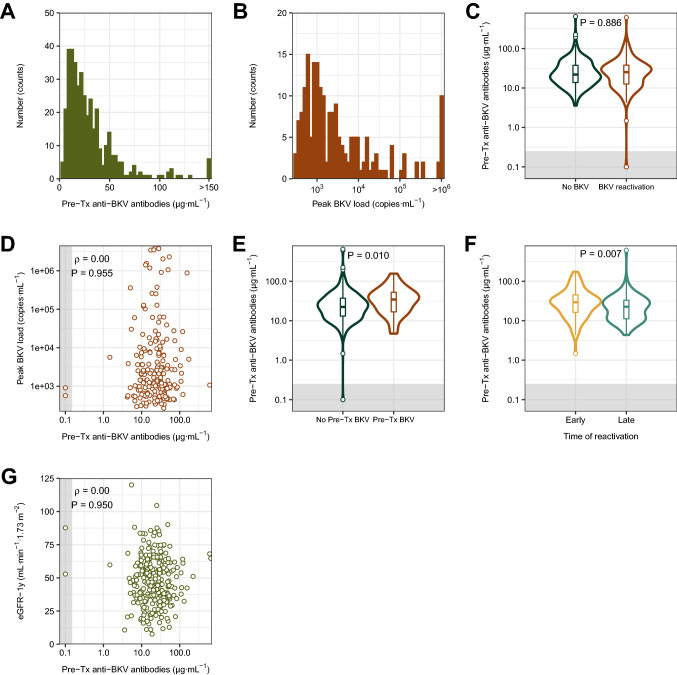
Pre-transplant reactive to the BKV VP1 are almost ubiquitous and are associated with pre-Tx BKV load and early viral reactivation. **A** Histogram of the distribution of pre-transplant anti-BKV antibodies. **B** Histogram of the distribution of peak BKV viral load during the first post-transplant year among patients with viral reactivation. Note the logarithmic scale of the *x* axis. **C** Comparison of pre-transplant anti-BKV antibody levels between patients with no BKV reactivation during the first post-transplant year and patients with BKV reactivation. Note the logarithmic scale of the *y* axis. **D** Scatterplot of anti-BKV antibody levels and peak BKV viral load during the first post-transplant year among patients with viral reactivation. Note the logarithmic scale of both axes. **E** Comparison of pre-transplant anti-BKV antibody levels between patients with no pre-transplant viral load and patients with pre-transplant BKV load. Note the logarithmic scale of the *y* axis. **F** Comparison of pre-transplant anti-BKV antibody levels between patients with BKV reactivation at an early and late time point. The categories early and late were defined with respect to the median reactivation time of 62 days post-transplant. Note the logarithmic scale of the y axis. **G** Scatterplot of anti-BKV antibody levels and graft function one year post-transplant. Note the logarithmic scale of the *x* axis. *BKV* BK virus; *Pre-Tx* pre-transplant, *eGFR-1y* estimated glomerular filtration rate 1 year post-transplant

Our results show that, even though pre-transplant anti-BKV-VP1 antibodies are nearly ubiquitous, they do not protect against viral reactivation. In fact, patients with pre-transplant viral load had significantly higher antibody levels than those with no detectable viral load. This suggests that antibodies are a marker for virus levels in peripheral blood and probably also in tissue. Interestingly, while patients with a high antibody concentration did not suffer from more frequent or severe reactivations, early reactivation was significantly associated with higher antibody levels. This further supports the hypothesis of pre-transplant antibodies as a marker for virus reactivation levels. Intriguingly, our results agree with our previous work with other cohorts, suggesting a central role for T cell-mediated immune response in BKV clearing [5, 6]. Here, quantification of pre-transplant neutralizing antibodies, as well as antibodies reactive to other BKV antigens, could contribute to the elucidation of the interplay between humoral immunity, cellular immunity and BKV.

In summary, our work demonstrates that pre-transplantation levels of BKV-specific binding antibodies cannot be employed to anticipate the post-transplant reactivation risk but might be used as a marker of pre-transplant viral load. Our data are in agreement with previously published studies, which showed that anti-BKV titres measured before transplantation in kidney recipients cannot be used as a predictive tool to manage clinical BKV infection [7–9]. However, measuring donor anti-BKV titres or the neutralizing capacity of the BKV-reactive antibodies in KTX rather correlates with a decreased risk of developing viremia and might be used to adapt pre-emptive therapies.

Further characterization of recipient and donor characteristics are needed to better understand the risk constellation of BKV reactivation and to implement effective prevention strategies.

BK virus, kidney transplantation, viral reactivations, risk assessment.

## Supplementary Information

Below is the link to the electronic supplementary material.

Supplementary file1 (DOCX 8284 kb)

## Data Availability

Limited data access requests can be sent to Nina Babel (nina.babel@charite.de).
