# Supplementary material for: Lack of predictive capacity of pre-transplant anti-BK virus antibodies for post-transplant reactivation
Source: J Nephrol. 2022 Dec 2;36(4):1071–3. doi: 10.1007/s40620-022-01487-7 (PMC10227150; doi:10.1007/s40620-022-01487-7)
Supplement: Supplementary file 1 — Supplementary file1 (DOCX 8284 kb) [file 40620_2022_1487_MOESM1_ESM.docx]

**Supplementary methods**

**Quantification of serum IgG antibodies against the structural BKV protein VP1**

96 well plates (Costar Assay Platte High Binding, Corning) were coated at 4°C overnight with 1 µg·mL^‑1^ of BKV-VP1 capsid protein of the AS strain (Abcam, ab74567), which was reconstituted in 100 µl sterile ddH_2_0 to a final stock concentration of 1 mg·mL^-1^. Plates were washed twice with 150 µl wash buffer (Phosphoprotein Detection Wash Buffer, Biorad), blocked with Roti-Block (Carl Roth GmbH) solution for 2 hours at 37°C to minimized unspecific bindings and washed for another two times with wash buffer. Patient serum was diluted 1:250 in Roti-Block solution. The standard curve was prepared using 1:2 serial dilutions of the monoclonal anti-human-BKP-VP1 antibody (Sigma Aldrich, sab1412996) with a starting concentration of 500 ng·mL^-1^.  100 µl of standard and diluted serum samples were then added onto the coated plate and incubated for 1 hour at room temperature. After washing twice, 100µl of 1 µg·mL^-1^ anti-human-IgG-HRP (Abcam, ab7499) diluted in PBS was added to each well and incubated for 1 hour at room temperature. After a final washing step, the plates were developed using the Substrate Reagent Pack (R&D systems) according to manufactures instructions. Absorbance at 450 nm was measured and anti-BKV-VP1 antibody serum concentrations were calculated by interpolating with the standard curves.

**Supplementary table**

**Table S1:** Patient demographic and clinical characteristics, and treatment details. Data represented in number (percentage) or median (interquartile range) and range.

| **Parameter** | **Measurement** | **Total (n=397)** |
| --- | --- | --- |
| Male sex | number (%) | 249 (62.7%) |
| Age at pre-Tx (years) | Median (IQR) | 56 [46-64] |
| Weight at pre-Tx (kg) | Median (IQR) | 77 [67.8-89.4] |
| BMI at pre-Tx (kg·m-2) | Median (IQR) | 25.87 [23-29.18] |
| Patient with previous transplant | number (%) | 378 (95.2%) |
| Living donor | number (%) | 56 (14.1%) |
| Age of donor (years) | Median (IQR) | 55 [47-65] |
| Expanded criteria donor | number (%) | 177 (44.6%) |
| Total HLA mismatches | Median (IQR) | 3 [2-4] |
| Cold ischemia time (min) | Median (IQR) | 628 [407-842] |
| Arm A (basiliximab + MMF + tacrolimus + corticosteroids) | number (%) | 142 (35.8%) |
| Arm B (basiliximab + MMF + tacrolimus) | number (%) | 131 (33%) |
| Arm C (rATG + MMF + tacrolimus) | number (%) | 124 (31.2%) |
| VGCV Prophylaxis | number (%) | 222 (55.9%) |
| Acute rejection | number (%) | 42 (10.6%) |
| Severe BK virus viremia (>10,000 copies·mL-1) | number (%) | 42 (10.6%) |
| Severe cytomegalovirus viremia (>10,000 copies·mL-1) | number (%) | 14 (3.5%) |
| GFR-2w (ml·min-1·1.73m-2) | Median (IQR) | 31.34 [17.39-44.23] |
| GFR-1y (ml·min-1·1.73m-2) | Median (IQR) | 45.72 [34.83-59.04] |

TX – transplantation; BMI – body mass index; MMF - mycophenolate mofetil; ATG - anti-thymocyte globulin; VGCV - Valganciclovir; GFR – glomerular filtration rate

**Supplementary figure**

**Supplementary figure 1: Representative anti-BKV antibody ELISA. (A)** Image of an anti-BKV antibody ELISA. In row A/B the standard curve is shown in duplicated wells with A11/12 and B11/12 being the blanks. In row C/D 1-11 serum samples from KTX were loaded. In C12/D12 only PBS was added to the wells. In the rows E/F/G/H the same samples were loaded as in A/B/C/D, but the BKV-VP1 was not coated onto the wells, to control for unspecific binding of serum components to the wells. **(B)** Absorption measured at 450 nm for the standard curve (S1-S10), the blank and 11 serum samples from KTX (D1-D11). **(C)** Blank subtracted absorption measured at 450 nm for the standard curve (S1-S10), and 11 serum samples from KTX (D1-D11). **(E)** Absorption measured at 450 nm for all KTX serum samples detected in wells, which were coated with BKV-VP1 (D1-D11) or were left uncoated (D1-D11 uncoated.
